# The enhanced association between mutant CHMP2B and spastin is a novel pathological link between frontotemporal dementia and hereditary spastic paraplegias

**DOI:** 10.1186/s40478-022-01476-8

**Published:** 2022-11-22

**Authors:** Yongping Chen, Gopinath Krishnan, Sepideh Parsi, Marine Pons, Veroniki Nikolaki, Lu Cao, Zuoshang Xu, Fen-Biao Gao

**Affiliations:** 1 Department of Neurology, University of Massachusetts Chan Medical School, Worcester, MA 01605 USA; 2 Department of Neurology, Lab of Neurodegenerative Disorders, and Rare Disease Center of West China Hospital, Sichuan University, Chengdu, 610041 China; 3 Department of Biochemistry and Molecular Biotechnology, University of Massachusetts Chan Medical School, Worcester, MA 01605 USA; 4 Present Address: Center for Systems Biology, Massachusetts General Hospital and Harvard Medical School, Boston, MA 02114 USA

**Keywords:** CHMP2B, ESCRT, Frontotemporal dementia, Hereditary spastic paraplegias, Spastin

## Abstract

Chromosome 3-linked frontotemporal dementia (FTD3) is caused by a gain-of-function mutation in *CHMP2B*, resulting in the production of a truncated toxic protein, CHMP2B^Intron5^. Loss-of-function mutations in spastin are the most common genetic cause of hereditary spastic paraplegias (HSP). How these proteins might interact with each other to drive pathology remains to be explored. Here we found that spastin binds with greater affinity to CHMP2B^Intron5^ than to CHMP2B^WT^ and colocalizes with CHMP2B^Intron5^ in p62-positive aggregates. In cultured cells expressing CHMP2B^Intron5^, spastin level in the cytoplasmic soluble fraction is decreased while insoluble spastin level is increased. These pathological features of spastin are validated in brain neurons of a mouse model of FTD3. Moreover, genetic knockdown of spastin enhances CHMP2B^Intron5^ toxicity in a *Drosophila* model of FTD3, indicating the functional significance of their association. Thus, our study reveals that the enhanced association between mutant CHMP2B and spastin represents a novel potential pathological link between FTD3 and HSP.

**Supplementary Information:**

The online version contains supplementary material available at 10.1186/s40478-022-01476-8.

## Introduction

Frontotemporal dementia (FTD), associated with progressive atrophy of the frontal and/or temporal lobes of the brain, is the second most common form of dementia before 65 years of age [17]. FTD is characterized by progressive deterioration in social behavior, personality and language, and regarded as part of the spectrum disorder with the motor neuron disease amyotrophic lateral sclerosis (ALS). In particular, genetic mutations in a number of genes can cause both FTD and ALS, suggesting common pathogenic molecular mechanisms [6, 8]. Among them, mutations in charged multivesicular body protein 2B (*CHMP2B*) are especially interesting, as they are highly pathogenic in FTD linked to chromosome 3 (FTD-3) [19] and also found in some ALS cases [4, 18] and patients with early-onset Alzheimer’s disease (AD) [9].

*CHMP2B* encodes a subunit of the endosomal sorting complex required for transport III (ESCRT-III) complex whose molecular function was first shown to be essential during the formation of multivesicular bodies (MVBs) [2]. ESCRTs also play key roles in other cellular processes such as cytokinesis, virus budding, nuclear membrane repair, and autophagy [16]. In FTD3, a splicing site mutation in *CHMP2B* results in a C-terminal truncation of the protein missing the Microtubule Interaction Motif (MIM), named CHMP2B^Intron5^ [19]. A series of cell biology studies indicate that this mutant CHMP2B protein exhibits enhanced association to its binding partner CHMP4B and blockage in ESCRT-III disassembly [10, 11], leading to compromised endosomal functions [13, 22, 23, 25] and autophagy defects [10, 11, 14]. It remains to be identified what other cellular and molecular pathways are affected by CHMP2B^Intron5^.

Spastic paraplegia 4 (SPG4), the most common autosomal dominant form of hereditary spastic paraplegias (HSP), is caused by loss of function mutations in the *SPAST* gene that encodes spastin, a member of microtubule severing protein [5, 20, 21]. SPG4 patients show symptoms of clinical dementia but the underlying mechanisms remain unclear [26]. In this study, we find that spastin associates with greater affinity to CHMP2B^Intron5^ than to wildtype CHMP2B, revealing a novel potential pathological link between FTD and HSP.

## Materials and methods

### Mice and genotyping

The *tTA:CHMP2B*^*Intron5*^ and *tTA:CHMP2B*^*WT*^ mice used in this study have been described [7]; both males and females were used. All procedures involving mice were approved by the Institutional Animal Care and Use Committee at the University of Massachusetts Chan Medical School.

#### *Drosophila* genetics

Flies were maintained on a 12-h light/12-h dark cycle on standard cornmeal-yeast agar medium at 25 °C. *UAS-CHMP2B*^*Intron5*^ flies used were described previously [1]. *GMR-Gal4*, *UAS-RNAi SPAST* (#27,570), and *UAS-RNAi_SPAST* (#53,331) fly lines were from the Bloomington *Drosophila* Stock Center. For genetic interaction studies, the recombined fly line (*GMR-Gal4*:*UAS-CHMP2B*^*Intron5*^) was crossed with *UAS-RNAi_SPAST* flies. To quantify the retinal degeneration phenotype, we classified the eye phenotype, with or without *SPAST* downregulation, into three groups: severe (+ + +), medium (+ +), and weak ( +). This classification was based on the relative abundance of black spots on the eye, ranging from a dozen or so scattered spots ( +) to spots covering approximately 50–70% or more of the eye surface (+ + +).

### Mammalian cell culture, siRNAs, constructs, transfection and immunoprecipitation

HEK293 and HeLa cells were cultured in Dulbecco’s modified Eagle’s medium (DMEM, Sigma) supplemented with 10% fetal calf serum (Life Technologies) and maintained in a humidified incubator at 37 °C with 5% CO_2_. All siRNAs for gene silencing were from Qiagen (Additional File 4: Table S1). *pCMV-3/FLAG-CHMP2B*^*Intron5*^ and *pCMV-3/FLAG-CHMP2B*^*WT*^ plasmids were generated as described [10]. Full-length human spastin M87 plasmids were generated by cutting the pCMV-Tag 3A/WT myc-M1 (Addgene, Cat. no. 87719) and pCMV-Tag 3B/WT myc-M87 (Addgene, Cat. no. 87722) and then subcloned into the pEGFP-C1 vector (Addgene). Full length spastin M87 is used throughout this study. siRNAs or constructs were transiently transfected into cells with RNAiMAXor Lipofactmine3000 (Invitrogen), as recommended by the manufacturer, for 48 h.

Three 100-mm tissue culture dishes of HEK293 cells at 70% confluency were transfected with pCMV-3/FLAG-CHMP2B^Intron5^, pCMV-3/FLAG-CHMP2B^WT^, or pCMV-3/FLAG empty vector with Lipofectamine 3000. After 48 h, transfected cells were collected and homogenized in immunoprecipitation (IP) lysis buffer (Thermo Fisher, Cat. no. 87787) with protease and phosphatase inhibitors (CST, catalog no. 5872). Homogenates were centrifuged at 4 °C for 10 min at 13,000g, to obtain supernatants. Protein concentrations of supernatants were determined with the Bradford assay (Bio-Rad). For co-IP experiments, supernatants of CMV-3/FLAG-CHMP2B^Intron5^, pCMV-3/FLAG-CHMP2B^WT^, or pCMV-3/FLAG with the same amount of total proteins were preabsorbed with anti-FLAG M2 affinity gel (Sigma, catalog no. A2220), incubated overnight at 4 °C, centrifuged and washed three times for 5 min each with washing buffer (50 mM Tris–HCl, pH 7.4, and 150 mM NaCl), and suspended in FLAG elution solution (Sigma catalog no. F4799) for 30 min at 4 °C. The supernatants were used for western blot.

### Proteomic analysis of CHMP2B^Intron5^ interacting proteins

To identify proteins that interact with CHMP2B^Intron5^, proteins in experimental and control IP samples were electrophoresed a short distance into a polyacrylamide–sodium dodecyl sulfate gel and stained with the Coomassie Brilliant Blue (Bio-Rad). In-gel digestion and liquid chromatography–tandem mass spectrometry analysis were done by the Mass Spectrometry Facility at the University of Massachusetts Chan Medical School. Protein abundance was estimated with IBAQ quantification, in which summed peptide intensities are normalized to the number of theoretically observable peptides of the protein. pCMV-3/FLAG served as a control to exclude nonspecific interacting proteins. Interacting proteins that were not associated with FLAG proteins but bound more to FLAG-CHMP2B^Intron5^ than FLAG-CHMP2B^WT^ were selected for further analyses. Total proteins were further ranked by iBAA value from most to least abundant. Mass spectrometry (MS) analysis was done by the UMass Chan Medical School Mass Spec Core with a standard protocol as published before [12].

### Western blots

The mouse cortex was dissected, quickly frozen at  − 80 °C, homogenized, and sonicated in RIPA buffer with proteinase and phosphatase inhibitors (CST, catalog no. 5872). The cultured cells were lysed in RIPA buffer (Thermo Scientific). The protein extract was centrifuged to remove tissue debris, and boiled for 5 min. Protein (20 μg) from each sample was subjected to SDS-PAGE using 4–20% precast gels (Bio-Rad) and immunobloted with the following primary antibodies: rabbit anti-spastin (Proteintech, catalog no. 22792–1-AP; 1:1000) and mouse anti-β-actin (Sigma-Aldrich, catalog no. A2228; 1:3000), overnight at 4 °C. After incubation, immunoblots were washed and incubated with IRDye fluorescent anti-rabbit and anti-mouse secondary antibodies (LI-COR Biosciences). Images were acquired with a LI-COR CLx Odyssey System.

### Subcellular fractionation and solubility analysis

HEK293 cells were collected 48 h after transfection and subjected to subcellular fractionation with a ProteoExtract Subcellular Proteome Extraction Kit (Millipore, catalog no. 539790), according to the manufacturer’s protocol for adherent cells. If some cells became nonadherent during the protocol, the cytosolic, membrane, and nuclear fractions were spun at 750 g, 5500 g, and 6800 g, respectively, for 10 min at 4 °C, to remove any contamination from later fractions. Proteins were resolved by SDS–PAGE and immunoblotted with spastin antibody (Proteintech, catalog no. 22792–1-AP; 1:1000).

For SPAST solubility analysis, cells were seeded into six-well dishes at 250,000 cells/well; 48 h after transfections, cells were washed with PBS, released with 0.25% trypsin, and resuspended in DMEM pre-warmed to 37 °C. The cells were then spun down, washed with PBS, and resuspended in 20 μl of PBS. The cells were lysed by two cycles of flash freezing on dry ice and rapidly thawing at 42 °C. The lysate was spun at 1000 g, and the resulting supernatant was transferred to a new tube and re-spun to remove any insoluble material. The pellet was rinsed 3 times with PBS and resuspended in the corresponding volume of supernatant and briefly sonicated with a tip sonicator (Sonopuls, catalog no. 2070). Equivalent fractions of total volume for 100 ng of supernatant and resuspended pellet were boiled with SDS loading buffer (50 mm Tris–Cl, pH 6.8, 2% (2 w/v) SDS, 0.1% (w/v) bromophenol blue) and 10 mm dithiothreitol, separated by SDS-PAGE on 10% polyacrylamide–sodium dodecyl sulfate gels and immunoblotted with spastin antibody (Proteintech, catalog no. 22792-1-AP; 1:1000).

### Immunofluorescence analysis of cultured cells

HeLa cells were fixed in 4% paraformaldehyde for 15 min, permeabilized with 0.3% Triton X-100 for 5 min, blocked with 5% bovine serum albumin for 30 min, and incubated overnight with the following primary antibodies: rabbit anti-spastin (Proteintech, catalog no. 22792–1-AP; 1:200), mouse anti-FLAG (Sigma, catalog no. F1804; 1:1000), rabbit anti-p62 (Proteintech, catalog no. 18420–1-AP; 1:2000). After incubation, the cells were washed three times with PBS, incubated first with donkey anti-mouse Alexa Fluor 488 secondary antibody (Invitrogen, catalog no. A-21202; 1:500) and then with goat anti-rabbit Alexa Fluor 568 secondary antibody (Invitrogen, catalog no. A-11011; 1:500) for 1 h at room temperature, and mounted with HardSet Mounting Medium with DAPI (Vectashield, catalog no. H-1500). Confocal images were acquired with a ZEISS LSM 800 laser-scanning confocal microscope and processed with ZEISS ZEN microscope software. Fluorescence images were acquired with a ZEISS inverted microscope (LSP T PMT).

### Immunostaining of mouse brain sections

Paraffin-embedded tissue sections were deparaffinized and hydrated in a series of graded alcohols. After antigen retrieval with citrate buffer (Sigma, C9999), the sections were washed once with water, treated with BLOXALL Endogenous Blocking Solution (Vector Lab, SP-6000–100) for 10 min washed with PBST for 10 min, blocked with Dako blocking reagent for 24 h, and incubated overnight with guinea pig anti-p62 (Progen, catalog no. GP62-C) and polyclonal anti-SPAST (Proteintech, catalog no. 22792) and 0.1% Triton-X 100; the antibodies were diluted 1:200 in DAKO antibody diluent (Agilent, S302283-2) overnight. The sections were washed three times with PBST for 10 min each and incubated with Alexa-conjugated secondary antibodies (Invitrogen, catalog nos. A-11075 and A32790) in detergent-supplemented DAKO antibody diluent buffer for 2 h in the dark. The sections were washed three times with PBST for 10 min each and mounted with DAPI Fluoromount-G Mounting Medium (Invitrogen). The total surface of stained brain sections from three mice per genotype group was scanned (Sanderson Center for Optical Experimentation) (SCOPE) (UMass Chan Medical School). Images from each channel were exported with TissueFACSL viewer software and processed in ImageJ. JACop plugin in Image J was used to calculate Mander’s overlap coefficient [3, 15]. P62 was considered as an aggregate marker to reveal the extent to which two signals occupy the same place. Manual thresholding was applied to exclude the background signals from all images. Representative figures were obtained with a confocal microscope (Leica SP8).

## Results and discussion

**Fig. 1:**
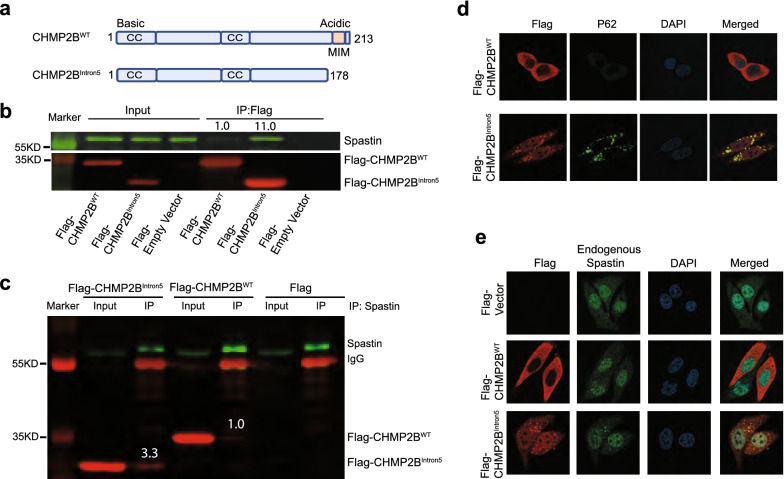
Increased biochemical interaction between spastin and CHMP2B^Intron5^. **a** Diagram of CHMP2B^WT^ and CHMP2B^Intron5^. CC: coiled coil. MIM: Microtubule Interaction Motif. **b** Proteins that coimmunoprecipitated with FLAG antibody were analyzed by western blots with spastin antibody. The experiment was repeated 4 times. After normalizing against the relative abundance of CHMP2B^WT^ versus CHMP2B^Intron5^, 11 times more spastin was bound to CHMP2B^Intron5^ than CHMP2B^WT^. **c** Immunoprecipitation with spastin antibody followed by western blot analysis with FLAG antibody. **d** and **e** Co-immunostaining analysis shows colocalization of p62 **d** and endogenous spastin **e** with CHMP2B^Intron5^ aggregates

The splicing site mutation in *CHMP2B* results in the production of a truncated protein missing the MIM domain, CHMP2B^Intron5^ (Fig. 1a), that is highly toxic when expressed in cultured cells and primary neurons [10, 22, 23]. To understand how mutant CHMP2B causes neurodegeneration through a gain-of-toxic function mechanism, we used immunoprecipitation (IP) and mass spectrometry to identify proteins that bind with greater affinity to CHMP2B^Intron5^ than to CHMP2B^WT^ in HEK293 cells (Additional File 1: Table S1). Among the top 12 interacting proteins were CHMP5, CHMP1B, and CHMP1A (Additional File 1: Fig. S1), other subunits of the ESCRT-III complex. We reported previously that CHMP2B^Intron5^ blocks dissociation of ESCRT-III [10, 11], thus, this result confirms the validity of this biochemical approach. Another protein that seems to associate with CHMP2B^Intron5^ stronger than to CHMP2B^WT^ is spastin (Additional File 1: Table S1), a microtubule-severing protein whose loss-of-function mutations are the most common genetic cause of hereditary spastic paraplegias (HSP) [20, 21]. We confirmed by IP and western blot analysis that spastin indeed binds with greater affinity to CHMP2B^Intron5^ than to CHMP2B^WT^ (Fig. 1b), as 11 times more spastin was pulled down by CHMP2B^Intron5^ than by CHMP2B^WT^ based on four independent experiments. This biochemical association was also confirmed by a reverse IP experiment in which spastin antibody pulled down 3.3 times more spastin-bound CHMP2B^Intron5^ than spastin-bound CHMP2B^WT^ based on three independent experiments (Fig. 1c). The lack of MIM in CHMP2B^Intron5^ indicates that its enhanced association with spastin may be mediated through other ESCRT-III components.

Expression of CHMP2B^Intron5^, but not CHMP2B^WT^, in HeLa cells resulted in the formation of p62-positive puncta (Fig. 1d), consistent with our previous observation that the p62 level in the insoluble fraction is greatly increased in neurons of *CHMP2B*^*Intron5*^ transgenic mice [7]. Interestingly, EGFP-tagged spastin was recruited to these cytoplasmic aggregates (Additional File 2: Fig. S2). More importantly, endogenous spastin also colocalized with CHMP2B^Intron5^ in these aggregates (Fig. 1e), further confirming the enhanced biochemical association between these two disease proteins. The C-terminal tail of CHMP1B, another ESCRT-III protein, directly interacts with the microtubule interacting and trafficking (MIT) domain of spastin [24, 27]. CHMP2B^Intron5^ prevents dissociation of ESCRT-III [10], thus, its enhanced associated with spastin may be mediated through other ESCRT-III components, such as CHMP1B. We speculate other ESCRT-III proteins that show an enhanced interaction with CHMP2B^Intron5^ versus CHMP2B^WT^ (Additional File 4: Table S1) may be also sequestered in p62/spastin-positive aggregates.

**Fig. 2:**
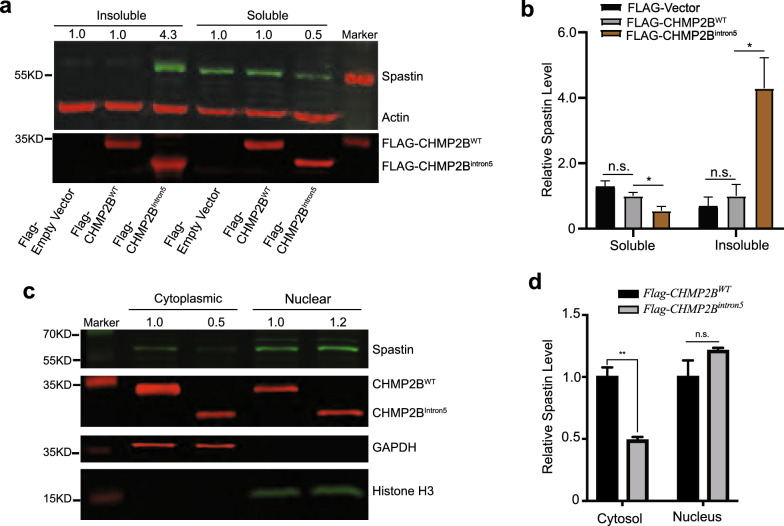
The effects of CHMP2B^Intron5^ on the solubility and subcellular localization of spastin. **a** The effect of CHMP2B^Intron5^ on the solubility of spastin in HEK293 cells, as shown by western blot analysis. **b** Relative abundance of spastin in soluble and insoluble fractions in cells expressing CHMP2B^WT^ or Flag-CHMP2B^Intron5^. Values are mean ± SEM, n = 4 independent experiments. n.s., not significant. *p < 0.05 by two-sided *t* test. **c** Western blot analysis of the subcellular localization of spastin in HEK293 cells expressing CHMP2B^Intron5^ or CHMP2B^WT^. **d** Quantification of the western blot in panel **c**. Values are mean ± SEM, n = 3. n.s., not significant. **p < 0.01, by two-sided *t* test

Like the p62 level in *CHMP2B*^*Intron5*^ mice, the spastin level in the insoluble fraction from cells expressing CHMP2B^Intron5^ was greatly increased than that in cells expressing CHMP2B^WT^ (Fig. 2a, b). As a consequence, the spastin level in the soluble fraction was decreased (Fig. 2a, b). This decrease was not due to reduced expression of *SPAST* mRNA (Additional File 3: Fig. S3). In fact, SPAST mRNA is increased by about 45% (Additional File 3: Fig. S3), which is probably a compensatory mechanism and further highlighting the decrease of spastin protein level in the soluble fraction is a direct consequence of CHMP2B^Intron5^ interaction. Spastin was localized in both the cytoplasm and the nucleus (Fig. 1e), but the level of soluble spastin was decreased only in the cytoplasm, as shown by fractionation and western blot analyses (Fig. 2c, d), consistent with the formation of cytoplasmic spastin aggregates (Fig. 1e). Thus, the increased aggregation of spastin and the decreased level of soluble spastin in the cytoplasm are novel pathological features of cellular toxicity induced by FTD3-associated mutant CHMP2B.

**Fig. 3:**
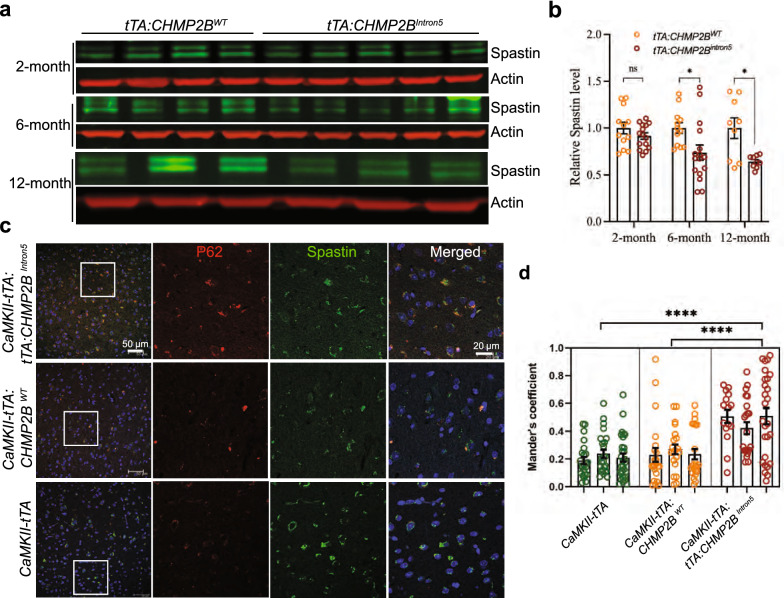
Functional significance of the interaction between spastin and CHMP2B^Intron5^ in a mouse model of FTD3. **a** Western blot analysis of the effect of CHMP2B^Intron5^ on spastin in a mouse model of FTD caused by mutant CHMP2B. The double bands are presumably two isoforms of spastin and the lower band corresponds to the M87 isoform that is used for transfection experiments throughout this study. **b** Level of total soluble spastin in the cortex of *tTA:CHMP2B*^*Intron5*^ mice. Values are mean ± SEM from three western blot experiments. *p < 0.05, **p < 0.01 by two-sided *t* test. **c** Representative images from identical areas of the cortex double-stained for p62 (red) and spastin (green). P62 is known to co-aggregate with CHMP2B^Intron5^ that is specifically expressed in excitatory neurons in this mouse model. The squares indicate areas shown at higher magnification in the adjacent panels. Scale bar: 20 µm. **d** Fraction of spastin signal overlapping with p62, calculated with Mander’s overlap coefficient. The analysis was done with the JACop plugin in Image J. *p < 0.05, **p < 0.01, ***p < 0.001 by one-way ANOVA and Bonferroni post hoc test for multiple comparisons

**Fig. 4:**
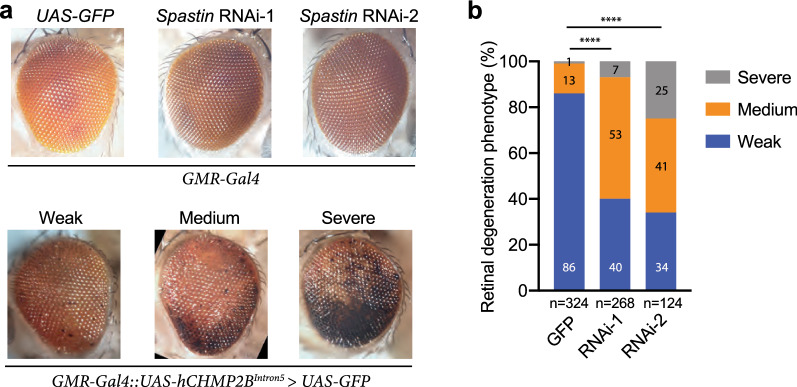
Functional significance of the interaction between spastin and CHMP2B^Intron5^ in a *Drosophila* model of FTD3. **a** Representative images of fly eyes with different genotypes. **b** Quantification of the retinal degeneration phenotypes in *CHMP2B*^*Intron5*^*-*expressing flies with or without *spastin* knockdown. The number of flies of each genotype is shown under the *x*-axis. The percentages of flies with severe, medium, or weak eye phenotypes are shown in the columns. ****p < 0.0001 by chi-square analysis

To further assess the functional significance of the biochemical interaction between CHMP2B^Intron5^ and spastin in vivo, we took advantage of our mouse model that expresses CHMP2B^Intron5^ specifically in forebrain excitatory neurons by *CAMKII* promoter controlled expression of tTA [7]. These mice exhibit FTD-like social behavioral deficits at 4 months, but not 2 months, of age, as well as cellular phenotypes such as ubiquitin-positive aggregates and astrogliosis [7]. We found that the level of soluble spastin was decreased in *CHMP2B*^*Intron5*^ mice as young as 2 months of age (Fig. 3a, b), suggesting an early disease phenotype, and this deficit was even more pronounced in older mice (Fig. 3a, b). In 12-month-old *CHMP2B*^*Intron5*^ mice, co-immunostaining analysis revealed the presence of spastin in p62-positive aggregates in mouse cortical neurons (Fig. 3c, d)—a novel pathological feature of FTD caused by *CHMP2B* mutations. Moreover, in a genetic interaction analysis in a *Drosophila* model of mutant CHMP2B toxicity [1], we found that RNAi knockdown of spastin with two different RNAi lines did not by itself cause retinal degeneration in the fly eye; however, it greatly increased CHMP2B^Intron5^ toxicity (Fig. 4), suggesting that partial loss of spastin function contributes to the toxicity of CHMP2B^Intron5^ in vivo.

Loss-of-function mutations in *SPAST* cause spastic paraplegia 4 (SPG4) [20, 21], the most common autosomal dominant form of HSP, which can be associated with clinical dementia [25]. *SPAST* mutations have also been reported in ALS [8]. The presence of spastin aggregates and the loss of soluble cytoplasmic spastin in FTD3 we identified in this study suggest that dysregulated association between CHMP2B and spastin may be a common novel pathogenic mechanism in HSP, amyotrophic lateral sclerosis, and FTD.

## Supplementary Information

**Additional file 1: Fig. S1.** The top 12 proteins that had a greater binding affinity for CHMP2B^Intron5^ than for CHMP2B^WT^, as shown by mass spectrometry analysis.

**Additional file 2: Fig. S2.** Immunocytochemical analysis of the interaction between CHMP2B and EGFP-Spastin in HeLa cells. Flag-CHMP2B^Intron5^ bound more EGFP-Spastin than Flag-CHMP2B^WT^. Scale bar, 10  μm.

**Additional file 3: Fig. S3.** Effect of CHMP2B^Intron5^ on the SPAST mRNA level in HEK293 cells in three independent experiments. ***p <0.001, ****p <0.0001, by two-sided *t* test.

**Additional file 4: Table S1.** Nucleotide sequences of SPAST RNAi.

## Data Availability

All data are available online after publication and materials can be shared upon request.
