# Supplementary material for: The enhanced association between mutant CHMP2B and spastin is a novel pathological link between frontotemporal dementia and hereditary spastic paraplegias
Source: Acta Neuropathol Commun. 2022 Nov 22;10:169. doi: 10.1186/s40478-022-01476-8 (PMC9682730; doi:10.1186/s40478-022-01476-8)
Supplement: Supplementary file 1 — Additional file 1: Fig. S1. The top 12 proteins that had a greater binding affinity for CHMP2BIntron5 than for CHMP2BWT, as shown by mass spectrometry analysis. [file 40478_2022_1476_MOESM1_ESM.pdf]

Proteins	Size (kDa)	Ratio	Molecular Function	Biological Process
CHMP5	25	$1.2x10^{7}$	ESCRT-III	MVB formation, autophagy, cytokinesis, virus budding etc.
CHMP1B	22	$2.9x10^{6}$	ESCRT-III	MVB formation, autophagy, cytokinesis, virus budding etc.
SLC3A2	58	$1.3x10^{6}$	double-stranded RNA binding, cadherin binding	amino acid transport, calcium ion transport
G3bp2	51	$1.9x10^{6}$	stress granule assembly	mRNA transport, stress granule-associated protein
RALA	24	$6.7x10^{5}$	Ras family small GTP binding protein	exocytosis, membrane raft localization
API5	57	$6.7x10^{5}$	RNA binding, Fibroblast growth factor binding	negative regulation of apoptotic process
Spastin	64	$5.2x10^{5}$	microtubule binding, beta-tubulin binding	microtubule bundle formation, regulation of cytokinesis
HRNR	282	$4.0x10^{5}$	calcium ion binding	cell envelop organization
FAF2	53	$4.8x10^{5}$	ubiquitin binding, lipase inhibitor activity	response to unfolded protein, lipid droplet organization
PDCD6IP	97	$3.3x10^{5}$	calcium depedent protein binding	viral budding via host ESCRT complex, apoptotic process
PIGS	62	$1.2x10^{5}$	glycosylphosphatidylinositol-anchor biosynthesis	metabolic pathways, post-translational protein modification
CHMP1A	22	23.3	ESCRT-III	MVB formation, autophagy, cytokinesis, virus budding etc.

kDa: kilodalton.

Ratio: the relative abundance of the protein that interacts with mutant CHMP2B versus wildtype CHMP2B.

This table represents three replicates.
