# Supplementary figures and images for: The enhanced association between mutant CHMP2B and spastin is a novel pathological link between frontotemporal dementia and hereditary spastic paraplegias
Source: Acta Neuropathol Commun. 2022 Nov 22;10:169. doi: 10.1186/s40478-022-01476-8 (PMC9682730; doi:10.1186/s40478-022-01476-8)

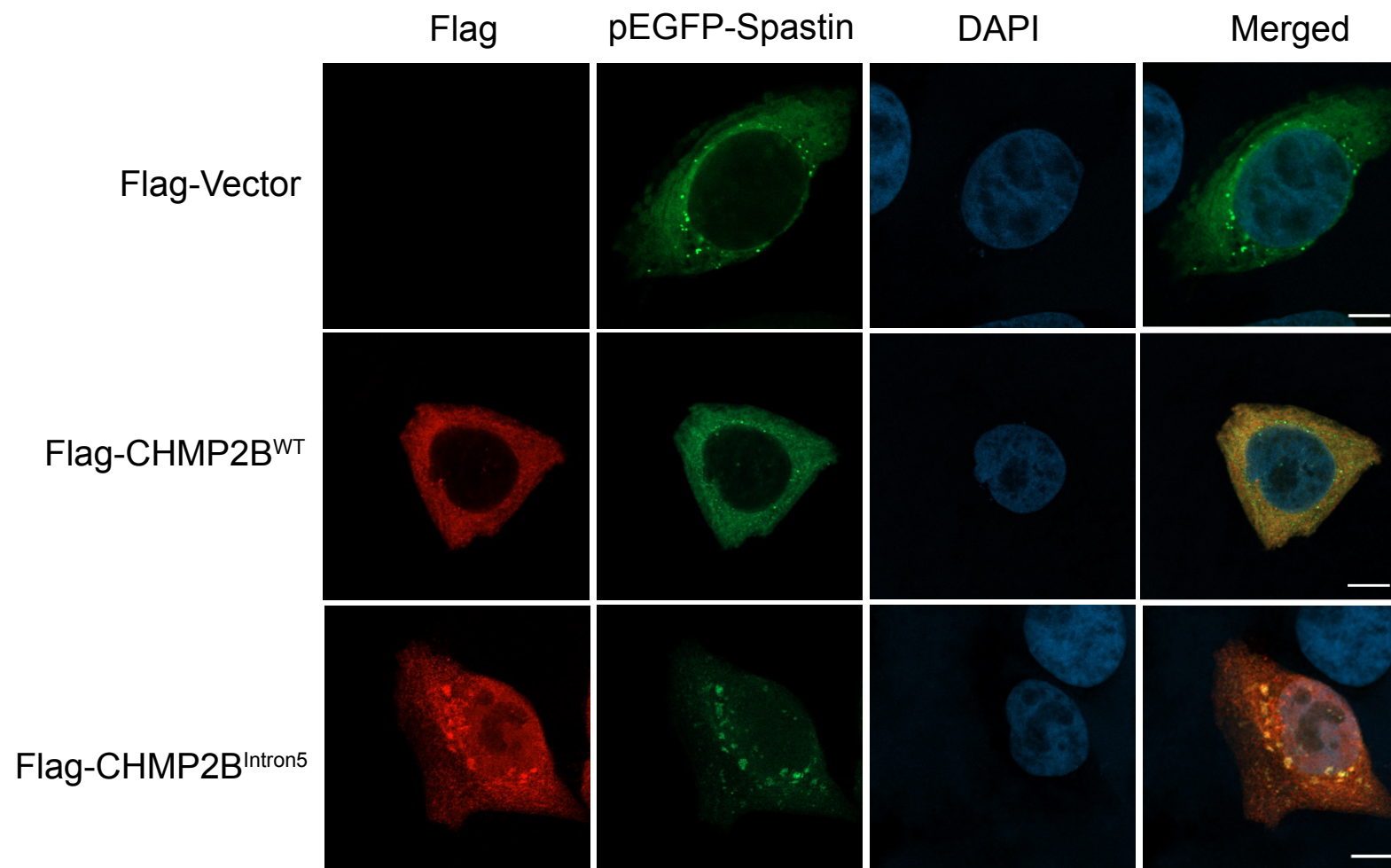

Supplement: Supplementary file 2 — Additional file 2: Fig. S2. Immunocytochemical analysis of the interaction between CHMP2B and EGFP-Spastin in HeLa cells. Flag-CHMP2BIntron5 bound more EGFP-Spastin than Flag-CHMP2BWT. Scale bar, 10 μm. [file 40478_2022_1476_MOESM2_ESM.pdf]

# Chen et al., Figure S3

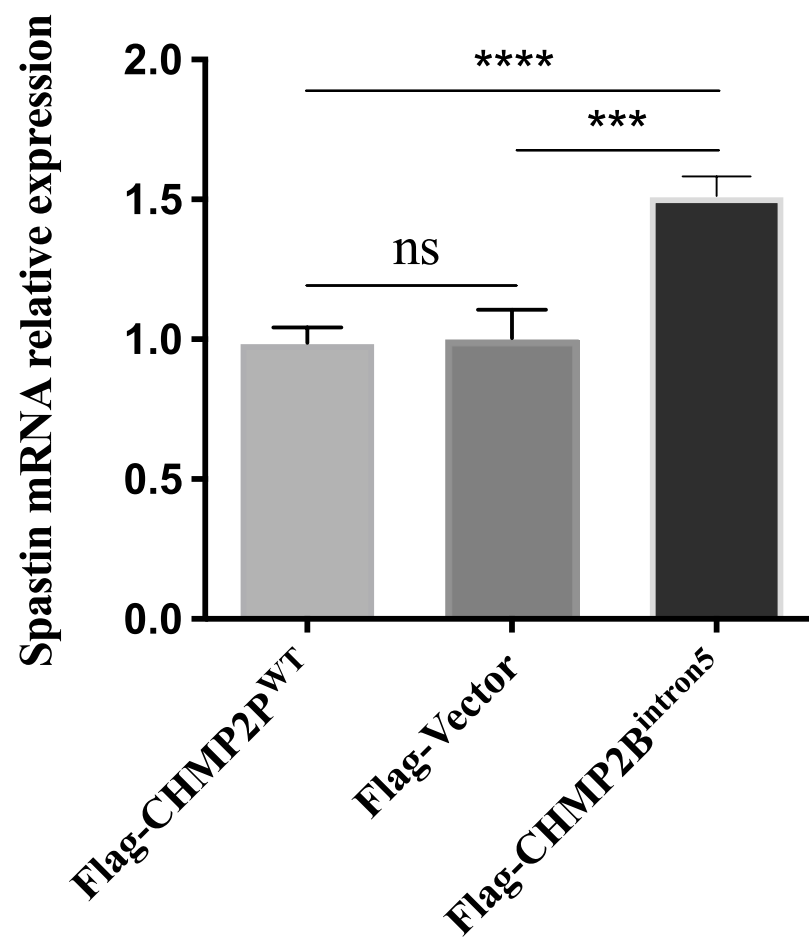

Supplement: Supplementary file 3 — Additional file 3: Fig. S3. Effect of CHMP2BIntron5 on the SPAST mRNA level in HEK293 cells in three independent experiments. ***p <0.001, ****p <0.0001, by two-sided t test. [file 40478_2022_1476_MOESM3_ESM.pdf]
