# Supplementary material for: The enhanced association between mutant CHMP2B and spastin is a novel pathological link between frontotemporal dementia and hereditary spastic paraplegias
Source: Acta Neuropathol Commun. 2022 Nov 22;10:169. doi: 10.1186/s40478-022-01476-8 (PMC9682730; doi:10.1186/s40478-022-01476-8)
Supplement: Supplementary file 4 — Additional file 4: Table S1. Nucleotide sequences of SPAST RNAi. [file 40478_2022_1476_MOESM4_ESM.pdf]

Table S1. The siRNA sequence of Spastin

No.	Catalog no.	Sequence
SPAST siRNA1	SI02781219	AACGTTATTGATACTTGGATA
SPAST siRNA2	SI03115119	TCCGCCCGAGTCGCCGCATAA
SPAST siRNA3	SI00108885	CACGATGCTAGTAGACGCCTA
SPAST siRNA4	SI00108892	TGCCCTTAGTTTACTGGTTAA
